# Enhancement of photosynthetic capacity in *Euglena gracilis* by expression of cyanobacterial fructose-1,6-/sedoheptulose-1,7-bisphosphatase leads to increases in biomass and wax ester production

**DOI:** 10.1186/s13068-015-0264-5

**Published:** 2015-05-30

**Authors:** Takahisa Ogawa, Masahiro Tamoi, Ayako Kimura, Ayaka Mine, Harumi Sakuyama, Eriko Yoshida, Takanori Maruta, Kengo Suzuki, Takahiro Ishikawa, Shigeru Shigeoka

**Affiliations:** Department of Advanced Bioscience, Faculty of Agriculture, Kinki University, 3327-204 Nakamachi, Nara, 631-8505 Japan; Core Research for Evolutional Science and Technology (CREST), Japan Science and Technology Agency (JST), Chiyoda-ku, Tokyo, 102-0076 Japan; Faculty of Life and Environmental Science, Shimane University, 1060 Nishikawatsu, Matsue, Shimane 690-8504 Japan; euglena Co., Ltd., 31F Iidabashi First Tower, 2-6-1 Koraku, Bunkyo-ku, Tokyo, 112-0004 Japan

**Keywords:** *Euglena gracilis*, Biofuel, Wax ester, Paramylon, Biomass, Fructose-1,6-/sedoheptulose-1,7-bisphosphatase, Photosynthesis

## Abstract

**Background:**

Microalgae have recently been attracting attention as a potential platform for the production of biofuels. *Euglena gracilis*, a unicellular phytoflagellate, has been proposed as an attractive feedstock to produce biodiesel because it can produce large amounts of wax esters, consisting of medium-chain fatty acids and alcohols with 14:0 carbon chains. *E. gracilis* cells highly accumulate a storage polysaccharide, a β-1,3-glucan known as paramylon, under aerobic conditions. When grown aerobically and then transferred into anaerobic conditions, *E. gracilis* cells degrade paramylon to actively synthesize and accumulate wax esters. Thus, the enhanced accumulation of paramylon through the genetic engineering of photosynthesis should increase the capacity for wax ester production.

**Results:**

We herein generated transgenic *Euglena* (*EpFS*) cells expressing the cyanobacterial fructose-1,6-/sedoheptulose-1,7-bisphosphatase (FBP/SBPase), which is involved in the Calvin cycle, to enhance its photosynthetic activity. FBP/SBPase was successfully expressed within *Euglena* chloroplasts. The cell volume of the *EpFS4* cell line was significantly larger than that of wild-type cells under normal growth conditions. The photosynthetic activity of *EpFS4* cells was significantly higher than that of wild type under high light and high CO_2_, resulting in enhanced biomass production, and the accumulation of paramylon was increased in transgenic cell lines than in wild-type cells. Furthermore, when *EpFS* cell lines grown under high light and high CO_2_ were placed on anaerobiosis, the productivity of wax esters was approximately 13- to 100-fold higher in *EpFS* cell lines than in wild-type cells.

**Conclusion:**

Our results obtained here indicate that the efficiency of biomass production in *E. gracilis* can be improved by genetically modulating photosynthetic capacity, resulting in the enhanced production of wax esters. This is the first step toward the utilization of *E. gracilis* as a sustainable source for biofuel production under photoautotrophic cultivation.

**Electronic supplementary material:**

The online version of this article (doi:10.1186/s13068-015-0264-5) contains supplementary material, which is available to authorized users.

## Background

The world is faced with an energy crisis due to the depletion of fossil fuels caused by growth of the global economy and population. Furthermore, the use of fossil fuels increases CO_2_ levels in the atmosphere. Therefore, feasible alternative energy sources are required as substitutes for fossil fuels. The production of biofuels from microalgae is now expected to become a practical solution for mitigating not only dependence on fossil fuels but also CO_2_ emissions. Microalgae are considered to be more advantageous than plants that are currently utilized as energy feedstocks due to their fast growth rates, high-lipid productivity, and cultivation on non-arable land areas, which does not compete with food production [1]. Therefore, extensive research has been conducted to identify useful microalgae all over the world and also improve biomass production through the metabolic engineering of known microalgae [2]. However, several problems such as the low productivity of microalgae and difficulties in consistently producing biomass at a large scale in highly variable outdoor culture conditions need to be overcome before microalgae can be used as an economically viable biofuel feedstock. To effectively improve microalgal biofuel production, the optimization of microalgal cultivation systems and the selection of species with many desirable biofuel traits are considered to be important. Furthermore, the development of genetic engineering for microalgae will be beneficial for enhancing biofuel productivity. Although genetic transformations of more than 30 different strains of microalgae have been reported [3], further research is still required to fully maximize microalgal biomass production for biofuels.

*Euglena gracilis*, a unicellular phytoflagellate, is widely distributed in freshwater and has been proposed as an attractive feedstock to produce biodiesel because it can produce wax esters with high productivity [4, 5]. *E. gracilis* cells highly accumulate a storage polysaccharide, a β-1,3-glucan known as paramylon, under aerobic conditions; therefore, these cells accumulate up to 50 % of paramylon per dry weight of the cells. When grown aerobically and then transferred into anaerobic conditions, *E. gracilis* cells degrade paramylon to actively synthesize and accumulate wax esters, consisting of medium-chain fatty acids and alcohols with 14:0 carbon chains being the main constituent (myristyl myristate). This phenomenon has been designated as wax ester fermentation due to the concomitant generation of ATP without any energy loss during the production of wax esters [4]. Myristic acid (C14:0) has more potential as a drop-in jet fuel than medium-length fatty acids such as palmitic acid (C16:0) and stearic acid (C18:0), because myristic acid is a component of kerosene used as jet fuel. In addition to the advantage of this resource as an alternative biofuel, *E. gracilis* is rich in nutrients such as vitamins, minerals, and well-balanced amino acids [6]; therefore, it is used as feedstock of dietary supplements, in the manufacture of cosmetics, and for the fortification of livestock feed. Thus, *E. gracilis* is one of the most attractive and ideal possibilities for biodiesel and biomass production. The genetic manipulation for *E. gracilis* not only increases its biomass production but also enriches its utility.

The metabolic engineering of CO_2_ assimilation promises to create useful *E. gracilis* strains capable of producing industrially feasible biodiesel. One of the strategies used to enhance algal productivity is optimization of the photosynthetic pathway. The rate-limiting enzymes involved in the Calvin cycle, fructose-1,6-bisphosphatase and sedoheptulose-1,7-bisphosphatase (FBPase and SBPase, respectively), are potential targets for the optimization of photosynthetic reactions [7, 8]. We previously reported that transgenic tobacco and lettuce plants overexpressing the cyanobacterial fructose-1,6-/sedoheptulose-1,7-bisphosphatase (*FBP/SBPase*) gene, which encodes a bifunctional enzyme having both FBPase and SBPase activities, in chloroplasts showed an enhanced CO_2_ assimilation rate and increased biomass production [7, 9, 10]. In order to achieve the objective of the present study, we first established transgenic *E. gracilis* cells expressing cyanobacterial FBP/SBPase and then showed that the introduction of the *FBP/SBPase* gene to microalgae improved biomass and wax ester production.

## Results

### Generation of transgenic *E. gracilis* cells expressing cyanobacterial FBP/SBPase

**Fig. 1:**
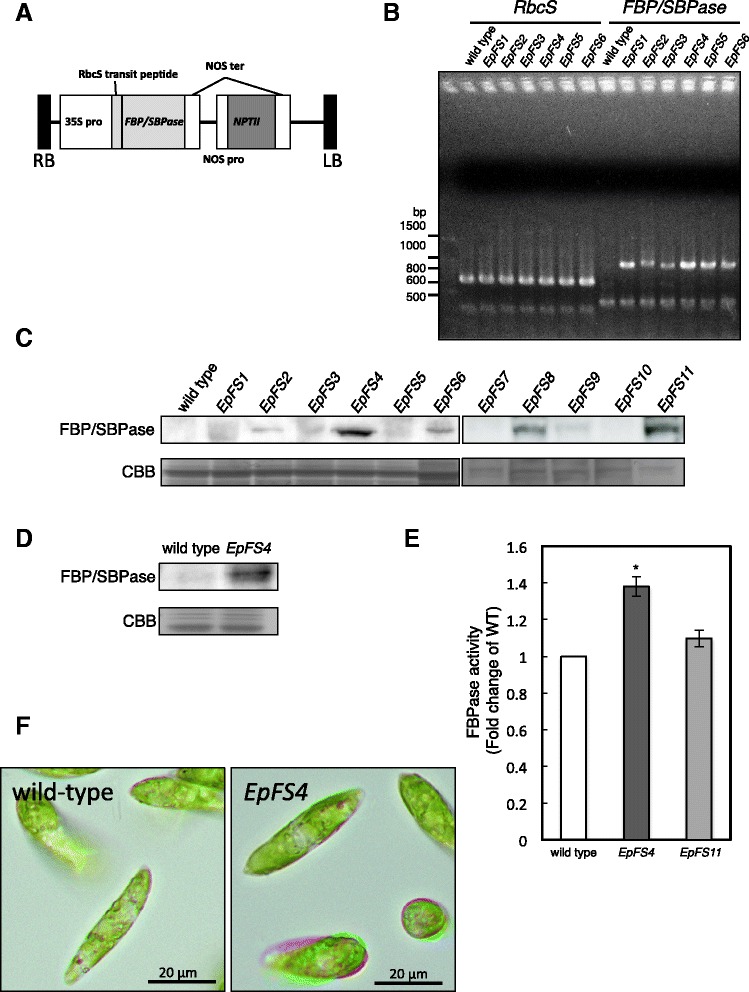
Isolation of transgenic *E. gracilis* cells having the *FBP/SBPase* gene. The construct structure of the gene using transformation of *Euglena* cells (**a**). Genomic PCR amplification of endogenous *rbcS* (637 bp) and *FBP/SBPase* (937 bp) genes from the wild-type and transgenic (*EpFS*) cell lines of *E. gracilis* (**b**). Western blot analysis of the crude extracts from wild-type and *EpFS* cell lines (**c**) and the intact chloroplastic fractions from wild-type and *EpFS4* cells (**d**) using an antibody raised against the FBP/SBPase protein. Total FBPase activity in the stationary phase wild-type and *EpFS* cell lines grown under normal conditions (**e**). The photographs of wild-type and *EpFS4* cells grown under normal conditions (**f**). Values are indicated as the mean ± standard deviation for three individual experiments. An *asterisk* indicates significant differences from the wild-type *E. gracilis* cells (*P < 0.05)

To generate transgenic *E. gracilis*, the cyanobacterial *FBP/SBPase* gene with a tomato rubisco small subunit (*rbcS*) transit peptide driven by the 35S promoter and neomycin phosphotransferase II (NPT II) gene as an antibiotic-resistant marker with the nopaline synthase (NOS) promoter was introduced into *E. gracilis* wild-type cells by microprojectile bombardment (Fig. 1a). Several paromomycin-resistant colonies were obtained after antibiotic selection, and these transgenic *E. gracilis* cells were named *EpFS* cells. Genomic DNA was isolated from wild-type and randomly selected six *EpFS* cell lines, and the transgene was analyzed by genomic PCR with gene-specific primers for the *FBP/SBPase* sequence. As shown in Fig. 1b, the expected 937-bp fragment was detected in all *EpFS* cell lines, but not in the wild-type cells. Immunoblot analysis using an anti-FBP/SBPase antibody showed that FBP/SBPase proteins were detected in the transgenic cell lines with the expected molecular size as mature proteins (approximately 40 kDa) (Fig. 1c). The *EpFS4* cell line accumulated the highest levels of the FBP/SBPase protein. FBPase activities were 1.4- and 1.1-fold higher in the *EpFS4* and *EpFS11* cell lines than in wild-type cells, respectively (Fig. 1e). The FBP/SBPase proteins were detected in chloroplasts isolated from the *EpFS4* cells (Fig. 1d). These results clearly indicated that the *FBP/SBPase* gene was successfully transformed into the *EpFS* cells, and the recombinant enzyme was then functionally expressed in the chloroplasts of *Euglena* cells after properly truncating the transit peptide.

### Photosynthesis, growth, and biomass production in EpFS cells under various growth conditions

**Fig. 2:**
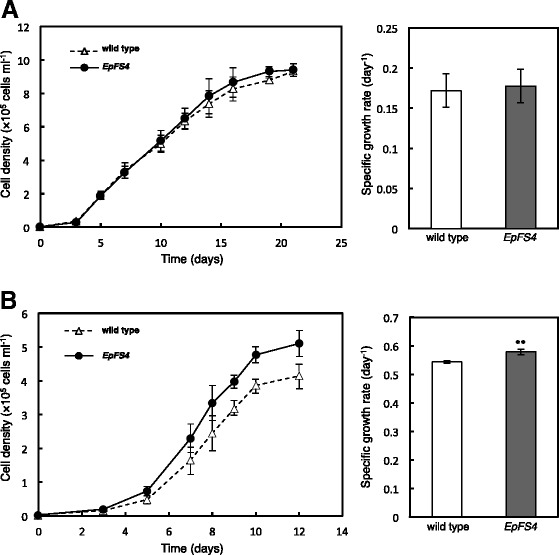
Growth curves and growth rates of wild-type and *EpFS4* cells grown under different cultivation conditions. Growth curves and growth rates of wild-type and *EpFS4* cells grown under normal (100 μmol photons m^−2^ s^−1^ at 0.04 % CO_2_) (**a**) and high light and high CO_2_ (350 μmol photons m^−2^ s^−1^ at 0.3 % CO_2_) (**b**), respectively. Values are indicated as the mean ± standard deviation for three to seven individual experiments. *Asterisks* indicate significant differences from the wild-type *E. gracilis* cells (**P < 0.01)

Cultivation conditions have been shown to greatly affect the growth and numerous chemical compositions of *Euglena* cells [11–13]. We determined whether the ectopic expression of FBP/SBPase in *Euglena* cells affected photosynthesis, growth, and biomass (dry weight) and paramylon production under various growth conditions. Under normal conditions (100 μmol photons m^−2^ s^−1^ at 0.04 % CO_2_), only slight enhancements were observed in photosynthetic activity in the *EpFS4* cells (data not shown). Concomitant with this, the cell volume of *EpFS4* was significantly larger than that of wild-type cells (Table 1). While, no differences were observed in morphological and cell growth between wild-type and *EpFS4* cells (Figs. 1f and 2a). Biomass and paramylon production was slightly higher in *EpFS4* cells than in wild-type cells (Additional file 1: Tables S1 and Additional file 2: Table S2).

**Table 1:** Cell volume of wild-type and *EpFS4* cells under different growth conditions

Light intensity	100 μmol photons m^−2^ s^−1^	350 μmol photons m^−2^ s^−1^	350 μmol photons m^−2^ s^−1^
CO_2_ conc.	0.04 %	0.04 %	0.30 %
Wild type	1626.9±25.9	2011.0±228.6	2980.9±414.6
*EpFS4*	1810.7±89.3^*^	2623.8±191.5^*^	3357.1±194.5^*^

Values are given in fl per cell and are the mean ± standard deviation of the analysis of three to seven independent cultures. *Asterisks* indicate significant differences from the wild-type *E. gracilis* cells in each growth conditions (*P < 0.05)

**Fig. 3:**
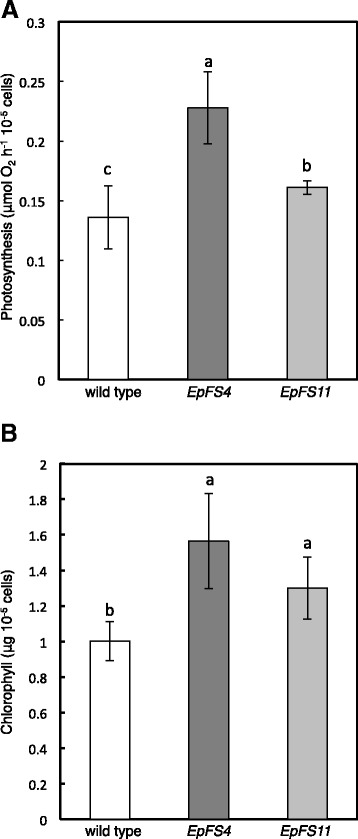
Photosynthetic activities and chlorophyll contents of wild-type and *EpFS* cell lines. The photosynthetic activities (**a**) and chlorophyll contents (**b**) of wild-type and *EpFS* cell lines grown under high light and high CO_2_. Values are indicated as the mean ± standard deviation for three to seven individual experiments. Different *letters* indicate significant differences (*P* < 0.05)

We then evaluated photosynthesis and the growth of *EpFS4* cells under high light (350 μmol photons m^−2^ s^−1^ at 0.04 % CO_2_) and/or high CO_2_ (0.3 % CO_2_). The growth of wild-type and *EpFS4* cells was markedly inhibited when grown under high light at 0.04 % CO_2_ (Additional file 3: Figure S1). The cell density of *EpFS4* line was higher than wild-type cells, although no significant differences were observed in the growth rates of wild-type and *EpFS4* cells (Additional file 3: Figure S1). Under high light and high CO_2_ (350 μmol photons m^−2^ s^−1^ at 0.3 % CO_2_), the high-light-induced inhibition of growth in both wild-type and *EpFS4* cells was markedly alleviated (Fig. 2b). Growth rate and cell volume were faster and larger in *EpFS4* cells than in wild-type cells, respectively (Fig. 2b, Table 1). Furthermore, the enhancement of photosynthetic activity and significant accumulation of chlorophylls were observed in *EpFS* cell lines under high light and high CO_2_ (Fig. 3a, b). The productions of paramylon were significantly greater in *EpFS* cell lines than in wild-type cells (Table 2). Biomass productions were also approximately 1.3- and 2.0-fold higher in transgenic cell lines than in wild-type cells (Table 3).

**Table 2:** Paramylon content in wild-type and transgenic cell lines grown under high light and high CO_2_

Genotypes	μg 10^−5^ cells	mg g^−1^ DW	Volumetric yield (mg l^−1^)
Wild type	38.4 ± 5.8 b	244.9 ± 19.6 b	79.7 ± 26.0 c
*EpFS4*	51.9 ± 0.6 a	283.4 ± 19.2 a	150.3 ± 19.0 a
*EpFS11*	49.6 ± 9.9 a	265.8 ± 20.6 a	116.2 ± 24.8 b

Values are the mean ± standard deviation of the analysis of 4 to 11 independent cultures. Different *letters* indicate significant differences (*P* < 0.05)

**Table 3:** Biomass production of wild-type and transgenic cell lines grown under high light and high CO_2_

Genotypes	mg of DW l^−1^
Wild type	321.1 ± 117.7 c
*EpFS4*	631.1 ± 89.9 a
*EpFS11*	430.3 ± 65.5 b

Values are the mean ± standard deviation of the analysis of five to eight independent cultures. Different *letters* indicate significant differences (*P* < 0.05)

### Wax ester production and fatty acid profiling

Because wax esters are synthesized by the degradation of paramylon in *E. gracilis*, the accumulation of paramylon may impact on their production. We evaluated the amount of a wax ester (myristyl myristate) and fatty acid content in wild-type and *EpFS* cell lines grown under normal conditions or high light and high CO_2_ and then placed onto anaerobiosis. After 24-h static culture under dark anaerobiosis, no significant difference was observed in the amount of the wax ester or fatty acid content between wild-type and *EpFS4* cells grown under normal conditions (Additional file 4: Table S3 and Additional file 5: Table: S4). The amounts of wax ester in *EpFS* cell lines grown under high light and high CO_2_ were approximately 13- and 100-fold higher, respectively, than those in wild-type cells after the anaerobic incubation (Table 4). In addition, total fatty acid content was higher in *EpFS* cell lines reflecting the increased levels of FBPase activities than in wild-type cells, especially myristic acid (C14:0), which was approximately 2.5- and 5-fold higher, respectively, than that in wild-type cells (Table 5).

**Table 4:** Wax ester (C28) content in wild-type and transgenic cell lines after anaerobic incubation

Genotypes	ng 10^−5^ cells	ng mg^−1^ DW
Wild type	0.6 ± 0.2 c	4.4 ± 1.5 c
*EpFS4*	65.2 ± 17.3 a	418.3 ± 92.0 a
*EpFS11*	10.4 ± 1.8 b	56.8 ± 11.3 b

The wild-type and transgenic cell lines grown under high light and high CO_2_ were placed on anaerobiosis for 24 h_._ Values are the mean ± standard deviation of the analysis of two to four independent cultures. Different *letters* indicate significant differences (*P* < 0.05)

**Table 5:** Fatty acid content in wild-type and transgenic cell lines after anaerobic incubation

Chain length	Wild type	*EpFS4*	*EpFS11*	Wild type	*EpFS4*	*EpFS11*
		(ng 10^−5^ cells)			(ng mg^−1^ DW)	
C12	15.2 ± 6.8 b	27.2 ± 2.9 a	29.1 ± 1.6 a	106.6 ± 38.0 b	176.9 ± 28.9 a	164.6 ± 19.6 a
C14	57.0 ± 5.3 c	318.9 ± 32.3 a	158.3 ± 33.3 b	424.7 ± 67.6 c	2072.8 ± 322.6 a	963.5 ± 372.3 b
C15	14.7 ± 1.8	11.3 ± 0.4	9.7 ± 2.7	105.6 ± 16.2	73.0 ± 6.6	49.6 ± 19.2
C16	256.6 ± 43.6 b	348.6 ± 10.3 a	301.5 ± 61.3 b	1828.2 ± 169.1 b	2260.5 ± 187.2 a	2040.3 ± 276.5 b
C18	46.1 ± 8.4	30.9 ± 2.5	29.7 ± 3.6	332.5 ± 71.5	200.4 ± 17.8	186.6 ± 50.0

The wild-type and *EpFS4* cells grown under high light and high CO_2_ were placed on anaerobiosis for 24 h_._ Values are the mean ± standard deviation of the analysis of three to six independent cultures. Different *letters* indicate significant differences (*P* < 0.05)

## Discussion

Critical matters that need to be resolved for the utilization of microalgae as a feasible energy source are their low productivity and the high-energy input required for their cultivation and processing. Bioprocess and metabolic engineering involving the introduction of appropriate genes appears to be a useful approach for overcoming some of these obstacles. We previously succeeded in generating high-yield transgenic tobacco and lettuce plants by introducing cyanobacterial FBP/SBPase into their chloroplasts [7, 9, 10]. We established transgenic *E. gracilis* expressing cyanobacterial FBP/SBPase to enhance its photosynthetic activity in the present study, thereby increasing its productivity of biomass and/or a wax ester. We transformed the cyanobacterial *FBP/SBPase* gene into wild-type *E. gracilis* cells and generated *EpFS* cell lines. The transgene and expression of the FBP/SBPase protein as its mature form, except for the transit peptide, were detected in the *EpFS* cell lines. *EpFS4* cells highly expressed mature FBP/SBPase in chloroplasts, and this was accompanied by a significant increase in FBPase activity. *EpFS* cell lines also showed phenotypes such as a larger cell volume and enhanced photosynthetic activity over wild-type cells, resulting in increased biomass and wax ester production under high light and high CO_2_. These results indicate that the cyanobacterial FBP/SBPase functioned in the transition of the rate-limiting step in the Calvin cycle of *E. gracilis* cells. However, several *EpFS* cell lines showed poor expression of FBP/SBPase protein (Fig. 1c). Poor expression of transgenes in *Chlamydomonas* has been reported; possible mechanisms for low expression of protein include gene silencing, inefficient transcription from heterologous promoters, improper RNA processing, mRNA instability, and instability of expressed proteins [3, 14]. Therefore, a variety of methods to improve the stability of transgene expression developed mainly with *Chlamydomonas reinhardtii* by the use of valuable endogenous promoters; inclusion of species-specific 5′, 3′, and intron sequences; and proper codon usage [3, 14, 15]. In this study, we used cauliflower mosaic virus 35S (CaMV35S) promoter and NOS terminator, generally used to express target genes*.* Therefore, it would be possible to refine the more stable expression of transgenes in *E. gracilis* after optimizing a variety of new genetic tools.

Although some enzymes involved in the Calvin cycle are present at levels well in excess of those required to sustain a continued rate of CO_2_ fixation, FBPase and SBPase levels are known to be markedly lower than those of other enzymes in the cycle [8]. In fact, the activity of FBPase in *E. gracilis* cells was extremely lower than those of NADP^+^-dependent glyceraldehyde 3-phosphate dehydrogenase (NADP^+^-GAPDH) and phosphoribulokinase (PRK) (Additional file 6: Table S5). Fang et al. recently reported that the Calvin cycle enzyme, SBPase from *C. reinhardtii*, was transformed into *Dunaliella bardawil*, and the transformant showed enhanced photosynthesis and increased glycerol biosynthesis [16]. These findings indicate that FBPase and SBPase in the Calvin cycle are important strategic positions that enhance the photosynthetic capacity of photosynthetic microalgae.

We previously reported that transgenic plants in which the capacity of the Calvin cycle was enhanced grew faster than wild-type tobacco plants under 400 μmol photons m^−2^ s^−1^, but not under 100 μmol photons m^−2^ s^−1^ [7, 9, 10, 17]. Photosynthetic activity was significantly higher in transgenic plants than in wild-type plants at irradiances above 200 μmol photons m^−2^ s^−1^. Under low light, photosynthetic CO_2_ assimilation was not limited by the capacity of the Calvin cycle but was by that of photosynthetic electron transport [18]. Accordingly, transgenic plants expressing FBP/SBPase showed the phenotype, except for under 100 μmol photons m^−2^ s^−1^. Clear phenotypes were also observed in *EpFS* cell lines under high light and high CO_2_. Under 100 μmol photons m^−2^ s^−1^, the volume of *EpFS4* cells was significantly higher (Table 1), while the growth rate was almost the same as that of wild-type cells (Fig. 2a). Under high light and high CO_2_, both the cell volume and growth rate of *EpFS4* cells were higher than those of wild-type cells (Fig. 2b, Table 1). On the other hand, the growth of wild-type and *EpFS4* cells was inhibited under high light at atmospheric CO_2_, although the cell density of *EpFS4* cells was higher than wild-type cells (Additional file 3: Figure S1). These results suggest that wild-type cells could not consume excess reducing energy from the photosynthetic electron transport system under high light at atmospheric CO_2_. One of the reasons for the tolerance of *EpFS4* cells to high light may be that these cells are able to consume an excess of reducing energy due to the enhanced capacity of the Calvin cycle. In support of this, the inhibition of growth in wild-type cells grown under high light was recovered by supplementation with high CO_2_ (Fig. 2b). These results suggest that the expression of FBP/SBPase leads to an increase in biomass production in *E. gracilis* cells under conditions in which the capacity of the Calvin cycle is a limiting factor for photosynthesis.

When *Euglena* grown aerobically is placed under anaerobic conditions, paramylon is rapidly converted into wax esters by wax ester fermentation [4]. A biochemical analysis has been conducted on the enzymes involved in the biosynthesis of fatty acids and wax esters in *Euglena* [5, 19, 20]; however, the regulatory mechanisms underlying the dynamic shift in wax ester fermentation remain unclear. Under high light and high CO_2_, the content of paramylon was significantly higher in *EpFS* cell lines than in wild-type cells, whereas no significant differences were observed in the content of the wax ester under aerobic conditions between wild-type and *EpFS* cells (data not shown). After 24-h static culture under dark anaerobiosis, the productions of the wax ester and myristic acid in *EpFS* cell lines grown under high light and high CO_2_ were greatly higher than that in wild-type cells (Table 4). In addition, increased levels of the wax ester and myristic acid between *EpFS4* and *EpFS11* were reflected in their FBPase activities. These results clearly indicated that the enhancement in photosynthetic capacity under aerobic conditions led to an increase in wax ester production under anaerobic conditions in *Euglena* cells. Previous studies identified pyruvate:NADP^+^ oxidoreductase as a rate limiting step in wax ester fermentation because its activity was inhibited by oxygen [21, 22]. These findings suggest that accumulation of paramylon and/or various metabolites, such as intermediates of the Calvin cycle, paramylon precursors, and pyruvate, as a substrate for pyruvate:NADP^+^ oxidoreductase, by enhancements in photosynthetic capacity, is one of the key metabolic factors for the biosynthesis of fatty acids and wax esters.

Inui et al. previously showed that all carbons in wax esters were supplied from paramylon, and approximately 40 % of paramylon was estimated to be converted to wax esters in *Euglena* cells heterotrophically grown in an organic carbon-rich medium [4]. In the present study, the amount of paramylon that accumulated under aerobic conditions was reduced by approximately 30 % after anaerobic incubation (Additional file 7: Table S6). However, the increase observed in wax ester production was not similar to the decrease in paramylon in wild-type and *EpFS4* cells photoautotrophically grown in an organic carbon-free medium. A recent metabolic profiling analysis in *Euglena* clearly indicated a dynamic shift in the central metabolic status including glycolysis, amino acid, and lipid production in response to aerobic/anaerobic conditions [13]. Therefore, organic carbons derived from paramylon are expected to be utilized not only for the synthesis of wax esters but also for numerous metabolic pathways in photoautotrophically grown cells under anaerobic conditions. When *Euglena* cells grow under heterotrophic conditions in an organic carbon-rich medium, the synthesis of wax esters from paramylon may be higher than that in photoautotrophically grown *Euglena* cells because the heterotrophic growth medium contained a large amount of available carbons for numerous metabolic pathways, such as amino acids and protein synthesis. Thus, elucidating the underlying regulatory mechanism and modulation of wax ester fermentation in photoautotrophically grown *Euglena* cells is needed for further increases in the production of wax esters by *E. gracilis* cells.

## Conclusions

In this work, we established transgenic *E. gracilis* expressing cyanobacterial FBP/SBPase to enhance its photosynthetic activity. The results of the present study demonstrated that enhancements in photosynthetic capacity facilitated biomass and wax ester production in photoautotrophically grown *E. gracilis*, and our strategy paves the way to increased photosynthesis and biomass production in a wide range of photosynthetic microalgae. Although further understanding of the mechanisms underlying wax ester biosynthesis and optimization of new genetic tools and cultivation systems for *E. gracilis* are required, this is the first step toward the utilization of *E. gracilis* as a sustainable source for biofuel production.

## Methods

### Cell strain and growth condition

*Euglena gracilis* Z (NIES-48) was cultured photoautotrophically in Cramer-Myers medium [23] on a rotary shaker (120 rpm) under continuous light (100 μmol photons m^−2^ s^−1^) at 26 °C.

### Plasmid construction

To generate transgenic *E. gracilis*, pRI101-AN, which has the NPT II gene cassette, was used to construct plasmids. Cyanobacterial FBP/SBPase fused with the transit peptide of the tomato *rbcS3C* gene was amplified by PCR from a previously reported plasmid [7] using the following primer set: 5′-*CATATG*GCTTCTTCAGTAATGTCC-3′ and 5′-*GAATTC*TTAACGGAGGCTAACCGTTTTGA-3′ (*Nde* I and *Eco*R I site italicized). The amplified DNA fragment was ligated into pT7Blue (Novagen, Madison, WI, USA) and then digested with *Nde* I and *Eco*R I. The *Nde* I-*Eco*R I DNA fragment was cloned into the *Nde* I-*Eco*R I sites of pRI101-AN, located downstream of the CaMV35S promoter, to generate pRI101-35S: *SL-rbcS-fbp/sbpase*. Regarding the transformation, the LB-RB region of pRI101-35S: *SL-rbcS-fbp/sbpase* containing the FBP/SBPase and NPT II genes was amplified by PCR using the following primer set: LB primer 5′-TGGCAGGATATATTGTAAACAAATTGACGC-3′ and RB primer 5′-GTTTACCCGCCAATAGTCAAACACTGATAG-3′. The amplified DNA fragments (approximately 4.8 Kbp) were purified and used for the transformation.

### Transformation of *E. gracilis* by microprojectile bombardment

Approximately 1 × 10^7^*E. gracilis* cells in the logarithmic growth phase were harvested by centrifugation (3000×*g*, 5 min, 25 °C), washed with 20 ml of distilled water, and resuspended in 2 ml of distilled water. The collected cells were placed onto 0.22-μm membrane filters (Millipore, Bedford, MA, USA) under a gentle vacuum, and the filters were then transferred onto CM agar plates. The cells were incubated at 26 °C for 24 h in the dark.

Regarding microprojectile bombardment, 0.25-μm gold nanoparticles (BBI Solutions, Cardiff, UK) were coated with the amplified DNA fragment. One milliliter of gold nanoparticle solution was centrifuged, washed twice with 1 ml of EtOH, and then washed once with 1 ml of distilled water. Gold nanoparticles were resuspended in 100 μl of distilled water and 6 μg of the amplified DNA fragment, 100 μl of 2.5 M CaCl_2_, and 40 μl of 0.1 M spermidine were added. The mixture was blended continuously for 20 min at 4 °C, 200 μl of EtOH was added, and the mixture was then centrifuged (10,000×*g*, 1 min, 4 °C). The pellet was washed three times with 200 μl EtOH and centrifuged for 1 min at 10,000×*g*. The DNA-coated nanoparticles were resuspended in 60 μl EtOH (6 μl shot^−1^) and maintained at 4 °C. The transformation of wild-type *E. gracilis* cells was performed using the Bio-Rad PDS-1000/He system (Bio-Rad, Hercules, CA, USA). DNA-coated nanoparticles were spread on a macrocarrier, which was placed in a macrocarrier holder. A rupture disc pressure of 900 psi and a target distance of 9 cm were used for bombardments. After microprojectile bombardment, the cells were washed off from the membrane filter with 2 ml of CM liquid medium and cultured for 24 h under continuous light. Cells were collected, plated onto a 1.5 % (*w*/*v*) agar CM plate (~1×10^5^ plate 1) containing 250 μg ml^−1^ paromomycin, and incubated at 26 °C under a continuous light intensity of 100 μmol photons m^−2^ s^−1^ until antibiotic-resistant colonies appeared. The transformed colonies were maintained on CM agar medium containing 250 μg ml^−1^ paromomycin.

### Genomic DNA extraction from *E. gracilis*

Cells were harvested and suspended in 5 ml of extraction buffer containing 50 mM Tris–HCl (pH 9.0), 100 mM NaCl, 10 mM EDTA, 2 % (*w*/*v*) SDS, and 200 μg ml^−1^ Proteinase K. After the cell suspensions were extracted with phenol/chloroform/isoamyl alcohol and treated with RNase A, genomic DNA was recovered by ethanol precipitation following phenol/chloroform/isoamyl alcohol treatments.

### PCR confirmation of transformation

Genomic PCR was performed with isolated genomic DNA (approximately 50 ng) as a template and the following primer sets: Eu rbcS-NF 5′-CTCTCATTACGATGCCATTTGAC-3′, Eu rbcS-631R 5′-GGTGTTGTCATTGGCGATGAAAGC-3′, 35S-60F 5′-TATCTCCACTGACGTAAGGG-3′, and FI-597R 5′-ACCATCGCTGATCAGACGG-3′. PCR amplification was performed for 40 cycles of 95 °C for 30 s, 55 °C for 30 s, and 72 °C for 60 s, followed by 72 °C for 10 min.

### Western blotting analysis

Proteins were separated by 10 % (*w*/*v*) NuPAGE® Gel (Invitrogen, Carlsbad, CA, USA) and blotted onto a PVDF membrane (GE Healthcare, Little Chalfont, UK) using the Xcell II™ Blot Module (Invitrogen, Carlsbad, CA, USA). The FBP/SBPase protein was detected using a mouse monoclonal FBP/SBPase antibody [24] and anti-mouse IgG–horseradish peroxidase (HRP) conjugate (Bio-Rad, Hercules, CA, USA) as the secondary antibody. Protein bands were detected using the enhanced chemiluminescence detection system (GE Healthcare, Little Chalfont, UK).

### Isolation of chloroplasts from *E. gracilis*

All procedures were conducted at 4 °C. *E. gracilis* cells were gently ground with a small amount of sea sand in 50 ml of cold grinding buffer containing 0.33 M sorbitol, 50 mM Tricine-KOH (pH 7.5), 1 mM MgCl_2_, 2 mM EDTA, and 10 mM NaCl. The homogenate was centrifuged at 300×*g* for 5 min. The resultant supernatant was centrifuged at 1500×*g* for 10 min to obtain the crude chloroplast fraction. Pelleted crude chloroplasts were resuspended in a small volume (~4 ml) of grinding buffer. The suspension was layered on top of the Percoll solution, which consisted of 20 ml of grinding buffer containing the 40 % (*v*/*v*) and 75 % (*v*/*v*) Percoll solutions, and centrifuged for 10 min at 2500×*g*. Intact chloroplasts were recovered as a band at the interface of the 40 % and 75 % Percoll layers. They were collected using a pipette, and the suspension was washed with grinding buffer (10 volumes of chloroplasts suspension) to remove Percoll. Pelleted chloroplasts were resuspended in a small volume of the rupture buffer containing 25 mM Tricine-KOH (pH 7.5), 1 mM MgCl_2_, 5 mM NaCl, and 0.5 % Triton X-100. Aliquots of fractions were subjected to Western blot analysis.

### Determination of chlorophyll content

Chlorophyll was extracted from *E. gracilis* cells with 80 % (*v*/*v*) acetone and measured by the method of Arnon [25].

### Measurements of cell density and cell volume

Stock cultures of wild-type and transgenic *E. gracilis* cells were grown in CM medium at stationary phase as a pre-culture, and 1.5 × 10^6^ cells were then placed into 1000-ml baffle-flasks containing 500 ml fresh CM medium. The cultures were grown under normal light (100 μmol photons m^−2^ s^−1^), high light (350 μmol photons m^−2^ s^−1^), and high light/high CO_2_ (350 μmol photons m^−2^ s^−1^, 0.3 % CO_2_) with mild agitation (120 rpm) at 26 °C in the plant growth chamber, BioTRON NC350 (Nippon Medical & Chemical Instruments Co., Ltd., Osaka, Japan). Cell density and volume were measured using the CASY Cell Counter and Analyzer System (Roche Applied Science, Basel, Switzerland). Specific growth rates (μ) were calculated from the slope of natural log-phase according to the formula: *μ* = ln (*N*_2_/*N*_1_)/(*t*_2_−*t*_1_), where *N*_1_ and *N*_2_ represent cell density at times *t*_1_ and *t*_2_ [26]. At the late log-phase, cells were collected by centrifugation and then freeze-dried to analyze the contents of paramylon, wax esters, and fatty acids. Anaerobiosis was applied by bubbling *N*_2_ to 30 ml of cells grown to the late log-phase, and anaerobic cells were then placed into the dark for 24 h.

### Assay of enzyme activities

*E. gracilis* cells (20 ml) in the late log-phase were harvested by centrifugation (8000×*g* for 5 min); resuspended in 100 mM Tris–HCl (pH 8.0) buffer containing 10 mM MgCl_2_, 1 mM EDTA, 2.5 mM DTT, 1 mM GSH, and 0.1 % (*v*/*v*) Triton X-100; and sonicated for a total of 1 min with five intervals of 10 s each. FBPase activity was assayed as described previously [7].

### Measurements of photosynthetic activity

The uptake and evolution of O_2_ were measured with an oxygen electrode (Hansatech Instruments Ltd., King’s Lynn, UK). The reaction mixture (2 ml) containing CM medium, 1 mM NaHCO_3_, and an arbitrary number of *E. gracilis* cells was illuminated (approximately 300 μmol photons m^−2^ s^−1^) with white light at 26 °C.

### Analysis of paramylon content

Approximately 10–20 mg of freeze-dried *E. gracilis* cells were suspended in 10 ml of acetone. The mixture was sonicated and vortexed for 1 min and then centrifuged at 11,000×*g* for 5 min. The supernatant was discarded, and these steps were repeated two times. After the pellet was resuspended with 10 ml of 1 % (*w*/*v*) SDS and vortexed for 1 min, the cell suspension was heated in boiling water for 30 min and centrifuged at 11,000×*g* for 15 min. After the supernatant was discarded, 1 ml of 0.1 % (*w*/*v*) SDS was added to the pellet and vortexed for 1 min. After centrifugation (11,000×*g* for 5 min), the pellet was washed with MilliQ water and resuspended in 10 ml of 1 N NaOH. To determine the amount of free glucose in the cell suspension, a 0.5-ml aliquot was mixed with 0.5 ml of 5 % phenol and 2.5 ml of concentrated H_2_SO_4_. After being incubated at 30 °C for 30 min, absorbance at 490 nm was determined. Absorbance was transformed to glucose equivalents using a standard calibration curve.

### Fatty acid and wax ester measurements

*Euglena* cells were harvested (1-ml medium) and fatty acid methyl esters (FAMEs) were prepared using a fatty acid methylation kit following the manufacturer’s instructions (Nacalai Tesque, Kyoto, Japan). The resulting FAMEs were then purified with a silica cartridge column and finally eluted with 3 ml of elution solution (n-hexane:methyl acetate = 94:2 (*v*/*v*)) supplied by the manufacturer (Nacalai Tesque, Kyoto, Japan). Extraction of the wax ester was performed using the method by Inui et al. [4] with slight modifications. Briefly, the collected cells were agitated vigorously with a 2.5-ml mixture of chloroform:methanol:water = 10:20:8 (*v*/*v*) and centrifuged to remove cell debris. The organic phase was dried with a rotary evaporator and resuspended in the elution solution. One microliter of each sample was analyzed using GCMS-QP2010 Ultra (Shimadzu, Kyoto, Japan) equipped with a DB-5ms capillary GC column (0.25 mm × 30 m, 0.25-μm thickness, Agilent, Santa Clara, CA, USA) coated with 5 % phenyl siloxane. The temperature program was as follows: the initial oven temperature was held at 60 °C for 0.5 min, then programmed to increase at 240 °C min^−1^ to 250 °C, and was held for 15 min. The carrier gas was helium with a constant linear velocity of 40 cm sec^−1^. The detector temperature was 250 °C. All data acquisition and processing were performed with the GC-MS Solution Software (Shimadzu, Kyoto, Japan).

### Data analysis

Significance of differences between data sets was evaluated by *t*-test. Calculations were carried out with Microsoft Excel software.

## Additional files

Additional file 1: Table S1. Paramylon content in wild-type and *EpFS4* cells grown under normal conditions. Additional file 2: Table S2. Biomass production of wild-type and *EpFS4* cells grown under normal conditions. Additional file 3: Figure S1. Growth curves and growth rates of wild-type and *EpFS4* cells grown under high light at 0.04 % CO_2_. Growth curves (left) and growth rates (right) of wild-type and *EpFS4* cells. Values are indicated as the mean ± standard deviation for three to seven individual experiments. Additional file 4: Table S3. Wax ester (C28) content in wild-type and *EpFS4* cells grown under normal conditions after anaerobic incubation. Additional file 5: Table S4. Fatty acid content in wild-type and *EpFS4* cells grown under normal conditions after anaerobic incubation. Additional file 6: Table S5. The FBPase, PRK, and NADP^+^-GAPDH activities in wild-type cells grown under normal conditions. Additional file 7: Table S6. Paramylon content in wild-type and *EpFS4* cells grown under different growth conditions after anaerobic incubation.

## Abbreviations

FBP/SBPase: Fructose-1,6-/sedoheptulose-1,7-bisphosphatase
FBPase: Fructose-1,6-bisphosphatase
SBPase: Sedoheptulose-1,7-bisphosphatase
rbcS: Rubisco small subunit
CaMV35S promoter: Cauliflower mosaic virus 35S promoter
NPT II: Neomycin phosphotransferase II
NOS: Nopaline synthase
NADP^+^-GAPDH: NADP^+^-dependent glyceraldehyde 3-phosphate dehydrogenase
PRK: Phosphoribulokinase
