# Supplementary material for: Enhancement of photosynthetic capacity in Euglena gracilis by expression of cyanobacterial fructose-1,6-/sedoheptulose-1,7-bisphosphatase leads to increases in biomass and wax ester production
Source: Biotechnol Biofuels. 2015 May 30;8:80. doi: 10.1186/s13068-015-0264-5 (PMC4459067; doi:10.1186/s13068-015-0264-5)
Supplement: Additional file 1: Table S1. — Paramylon content in wild-type and EpFS4 cells grown under normal conditions. [file 13068_2015_264_MOESM1_ESM.pdf]

Table S1 Paramylon content in wild-type and *EpFS4* cells grown under normal conditions

Genotypes	μg 10^{-5} cells	mg g^{-1} DW	Volumetric yield (mg l^{-1})
wild type	3.8±0.6	45.5±2.2	20.3±3.8
EpFS4	4.1±0.5	48.3±4.8	24.7±5.3

Values are the mean ± standard deviation of the analysis of 4-7 independent cultures.
