# Supplementary material for: Enhancement of photosynthetic capacity in Euglena gracilis by expression of cyanobacterial fructose-1,6-/sedoheptulose-1,7-bisphosphatase leads to increases in biomass and wax ester production
Source: Biotechnol Biofuels. 2015 May 30;8:80. doi: 10.1186/s13068-015-0264-5 (PMC4459067; doi:10.1186/s13068-015-0264-5)
Supplement: Additional file 2: Table S2. — Biomass production of wild-type and EpFS4 cells grown under normal conditions. [file 13068_2015_264_MOESM2_ESM.pdf]

Table S2 Biomass production of wild-type and *EpFS4* cells grown under normal conditions

genotypes	mg of D.W. l^{-1}
wild type	457.8±78.5
EpFS4	516.4±67.2

Values are the mean ± standard deviation of the analysis of 5-8 independent cultures.
