# Supplementary figures and images for: Enhancement of photosynthetic capacity in Euglena gracilis by expression of cyanobacterial fructose-1,6-/sedoheptulose-1,7-bisphosphatase leads to increases in biomass and wax ester production
Source: Biotechnol Biofuels. 2015 May 30;8:80. doi: 10.1186/s13068-015-0264-5 (PMC4459067; doi:10.1186/s13068-015-0264-5)

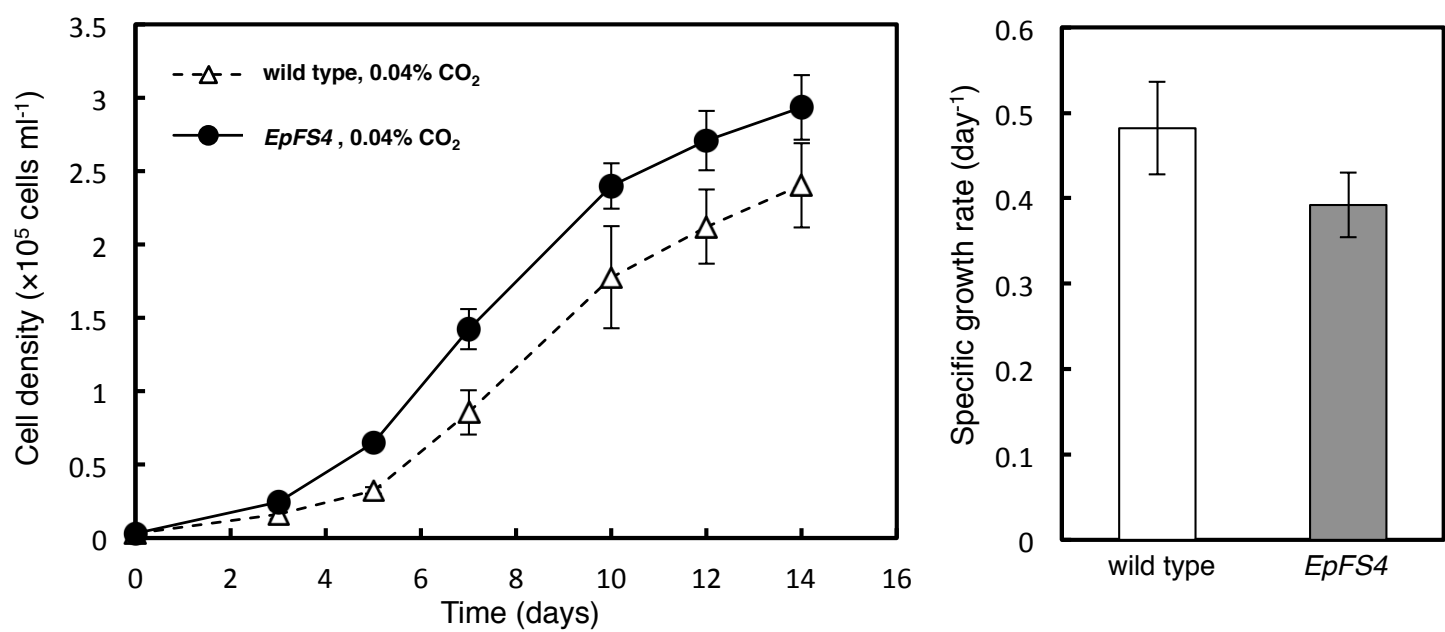

**Figure S1**

Supplement: Additional file 3: Figure S1. — Growth curves and growth rates of wild-type and EpFS4 cells grown under high light at 0.04 % CO2. Growth curves (left) and growth rates (right) of wild-type and EpFS4 cells. Values are indicated as the mean ± standard deviation for three to seven individual experiments. [file 13068_2015_264_MOESM3_ESM.pdf]
