# Supplementary material for: Enhancement of photosynthetic capacity in Euglena gracilis by expression of cyanobacterial fructose-1,6-/sedoheptulose-1,7-bisphosphatase leads to increases in biomass and wax ester production
Source: Biotechnol Biofuels. 2015 May 30;8:80. doi: 10.1186/s13068-015-0264-5 (PMC4459067; doi:10.1186/s13068-015-0264-5)
Supplement: Additional file 4: Table S3. — Wax ester (C28) content in wild-type and EpFS4 cells grown under normal conditions after anaerobic incubation. [file 13068_2015_264_MOESM4_ESM.pdf]

Table S3 Wax ester (C28) content in wild-type and *EpFS4* cells grown under normal conditions after anaerobic incubation

Genotypes	ng 10^{-5} cells	ng mg^{-1} DW
wild type	0.46±0.17	6.3±2.8
EpFS4	0.49±0.23	6.3±2.9

Values are the mean ± standard deviation of the analysis of 3-5 independent cultures.
