# Supplementary material for: Enhancement of photosynthetic capacity in Euglena gracilis by expression of cyanobacterial fructose-1,6-/sedoheptulose-1,7-bisphosphatase leads to increases in biomass and wax ester production
Source: Biotechnol Biofuels. 2015 May 30;8:80. doi: 10.1186/s13068-015-0264-5 (PMC4459067; doi:10.1186/s13068-015-0264-5)
Supplement: Additional file 5: Table S4. — Fatty acid content in wild-type and EpFS4 cells grown under normal conditions after anaerobic incubation. [file 13068_2015_264_MOESM5_ESM.pdf]

**Table S4** Fatty acid content in wild-type and *EpFS4* cells grown under normal conditions after anaerobic incubation

| Chain-length | wild type | *EpFS4* | wild type | *EpFS4* |
|---|---|---|---|---|
| | (ng $10^{-5}$ cells) | | (ng $mg^{-1}$ DW) | |
| C12 | 11.3±0.5 | 10.1±0.4 | 149.1±5.9 | 134.1±9.1 |
| C14 | 51.6±2.5 | 51.6±6.9 | 693.5±31.7 | 703.5±63.8 |
| C15 | 15.7±1.8 | 15.8±1.4 | 213.5±23.3 | 206.1±29.8 |
| C16 | 246.8±18.6 | 217.5±9.8 | 3240.7±18.6 | 2966.5±189.1 |
| C18 | 21.1±0.6 | 22.2±0.6 | 285.2±27.7 | 289.1±20.7 |

Values are the mean ± standard deviation of the analysis of 4-5 independent cultures.
