# Supplementary material for: Enhancement of photosynthetic capacity in Euglena gracilis by expression of cyanobacterial fructose-1,6-/sedoheptulose-1,7-bisphosphatase leads to increases in biomass and wax ester production
Source: Biotechnol Biofuels. 2015 May 30;8:80. doi: 10.1186/s13068-015-0264-5 (PMC4459067; doi:10.1186/s13068-015-0264-5)
Supplement: Additional file 6: Table S5. — The FBPase, PRK, and NADP+-GAPDH activities in wild-type cells grown under normal conditions. [file 13068_2015_264_MOESM6_ESM.pdf]

Table S5 The FBPase, PRK, and $NADP^+$-GAPDH activities in wild-type cells grown under normal conditions

	nmol min^{-1} 10^{-5} cells
FBPase	0.26±0.02
PRK	7.46±0.29
$NADP^+$-GAPDH	9.83±0.31

Values are the mean ± standard deviation of 3 determinations.
