# Supplementary material for: Enhancement of photosynthetic capacity in Euglena gracilis by expression of cyanobacterial fructose-1,6-/sedoheptulose-1,7-bisphosphatase leads to increases in biomass and wax ester production
Source: Biotechnol Biofuels. 2015 May 30;8:80. doi: 10.1186/s13068-015-0264-5 (PMC4459067; doi:10.1186/s13068-015-0264-5)
Supplement: Additional file 7: Table S6. — Paramylon content in wild-type and EpFS4 cells grown under different growth conditions after anaerobic incubation. [file 13068_2015_264_MOESM7_ESM.pdf]

**Table S6** Paramylon content in wild-type and *EpFS4* cells grown under different growth conditions after anaerobic incubation

| Growth conditions and genotypes | μg $10^{-5}$ cells |
|---|---|
| 100 μmol photons $m^{-2}$ $s^{-1}$, 0.04% $CO_2$ | |
| wild type | 3.4±0.2 |
| *EpFS4* | 3.5±0.4 |
| 350 μmol photons $m^{-2}$ $s^{-1}$, 0.30% $CO_2$ | |
| wild type | 32.5±7.8 |
| *EpFS4* | 33.3±8.4 |

Values are the mean ± standard deviation of the analysis of 5-7 independent cultures.
